# Erythropoiesis in Malaria Infections and Factors Modifying the Erythropoietic Response

**DOI:** 10.1155/2016/9310905

**Published:** 2016-02-29

**Authors:** Vrushali A. Pathak, Kanjaksha Ghosh

**Affiliations:** ^1^Department of Haematogenetics, National Institute of Immunohaematology (ICMR), KEM Hospital, Parel, Mumbai 400012, India; ^2^Surat Raktadan Kendra & Research Centre, Udhna Khatodara Urban Health Centre, Udhna Magdalla Road, Surat, Gujrat 395002, India

## Abstract

Anemia is the primary clinical manifestation of malarial infections and is responsible for the substantial rate of morbidity. The pathophysiology discussed till now catalogued several causes for malarial anemia among which ineffective erythropoiesis being remarkable one occurs silently in the bone marrow. A systematic literature search was performed and summarized information on erythropoietic response upon malaria infection and the factors responsible for the same. This review summarizes the clinical and experimental studies on patients, mouse models, and in vitro cell cultures reporting erythropoietic changes upon malaria infection as well as factors accountable for the same. Inadequate erythropoietic response during malaria infection may be the collective effect of various mediators generated by host immune response as well as parasite metabolites. The interplay between various modulators causing the pathophysiology needs to be explored further. Globin gene expression profiling upon malaria infection should also be looked into as abnormal production of globin chains could be a possible contributor to ineffective erythropoiesis.

## 1. Introduction

During malaria infection, anemia is a common complication and causes mortality and morbidity in patients, especially children and pregnant women [1]. In the malaria endemic areas, the coexistence of other circumstances like parasitic infestations, iron, folate, and Vitamin B12 deficiency, E-B virus (Epstein-Barr virus) infection, and aberrant immune response is the important considerations for anemia. Inherited red cell disorders like *α* and *β* thalassemia, sickle cell anemia, enzyme deficiencies, and membrane defects also interplay with the infection [2]. World Health Organization (WHO) recommended haemoglobin levels to diagnose anemia at sea level are <13.0 g/dL and <12.0 g/dL for adult men (age 15 years and above) and adult nonpregnant women (age 15 years and above), respectively [3].

The pathophysiology of malarial anemia is said to be complex and multifactorial [4] dependent on properties of both host and parasite. It is not only due to hemolysis of infected as well as uninfected erythrocytes but also due to inability to replenish erythrocytes lost by hemolysis through inadequate erythroid response.

During infection, there is obvious loss of infected erythrocytes through parasite maturation but many uninfected cells are also destroyed due to antibody sensitization or other physiochemical membrane changes and increased reticuloendothelial activity in spleen. Suppression of erythropoiesis has been said to be additional factor contributing to worsening the condition. Ineffective erythropoiesis (active erythropoiesis with premature death of red blood cells, a decreased output of erythrocytes from the bone marrow) and dyserythropoiesis (defective development of erythrocytes, such as anisocytosis and poikilocytosis) during malaria infection are profusely discussed topic and numerous clinical and experimental studies have been undertaken to demonstrate the same. Despite that, mechanism still remains unclear.

In this review, we summarized the four different approaches used by researchers to understand the erythropoiesis in malaria infections such as clinical evaluation of patients, in vivo mice studies, in vitro culture studies, and gene expression profiling as well as factors proposed or found to cause inappropriate erythropoietic response during infection.

## 2. Method

A systematic literature search was performed and we summarized information on erythropoiesis on malaria infections and factors causing inadequate erythropoiesis. We searched for peer-reviewed articles published in English language in the PubMed and ScienceDirect databases.

We used search terms like malaria, erythropoiesis, ineffective erythropoiesis, dyserythropoiesis, and hemozoin in title, abstract, and keywords.

Total 1604 records were identified through database searches. Among those 1368 were excluded after reading titles, abstract, and keywords. Only peer-reviewed articles were included and conference abstracts, proceedings, and project reports were excluded. 255 full-text articles were assessed for the relevance to the subject of review and 76 were included in the main study.

## 3. Results

### 3.1. Ineffective Erythropoiesis and Dyserythropoiesis

A variety of abnormalities in the number, morphology, and function of blood and bone marrow cells were observed in human as well as murine* Plasmodium* species like* P. falciparum*,* P. vivax*,* P. chabundi*,* P. berghei,* and* P. yoelii* [5, 6].

#### 3.1.1. Clinical Evaluation of Patients

Decreased production of erythroid cells was almost always found to be associated with dyserythropoiesis, that is, the production of morphologically defective cells which in functional terms results in ineffective erythropoiesis [4]. Light and electron microscope studies of marrow aspirates revealed morphological evidence of dyserythropoiesis in* P. falciparum* and* P. vivax* infected patients. Gambian patents infected with* P. falciparum* illustrated dyserythropoietic changes in the bone marrow [7]. Study on nine Thai* P. vivax* infected patients reported the presence of erythroblasts at various stages of degradation within the cytoplasm of macrophages [8]. Other changes as observed by light photomicrographs and electron micrographs included lymphocytosis in the bone marrow, appearance of giant metamyelocytes, macrophage hyperplasia, plasmacytosis, increased eosinophil granulocytopoiesis, reactive lymphocytes, monocytosis and mild neutrophilia in the peripheral blood, and increased number of megakaryocytes [8, 9]. Unlike* P. vivax* malaria, the microvasculature of the marrow was found to be obstructed by parasitized red cells in severe* P. falciparum* malaria [8]. Malarial anemia is characterized by the low reticulocytosis. It was first observed in 1939 by Vryonis [10] in acute malarial infections with* P. vivax and P. falciparum*. Srichaikul et al. [11] also reported the absence of reticulocytosis during malarial infections in which hemolysis was observed indicating transient suppression of erythropoiesis. The inadequate erythroid response was observed in spite of elevated levels of erythropoietin. Kurtzhals et al. [12] studied three patient categories for reticulocytosis. He used RDW as a surrogate marker of release of young erythrocytes and reticulocytes. Initially RDW was low in all the three categories, severe malarial anaemia (SA), cerebral malaria (CM), and uncomplicated malaria (UM), in spite of markedly increased concentrations of erythropoietin (EPO). As parasites were removed after treatment, RDW increased dramatically.

The reticulocyte production index (RPI) is also used as marker in the diagnosis of anemia as well as in the determination of erythropoietic response. It is a standard measure of reticulocyte production that corrects for both the degree of anemia and the early release of reticulocytes from the bone marrow in anemic patients [13]. In the study of 106 Kenyan children, erythropoietic suppression (RPI < 2.0) was observed in significantly more number of children in each of the three groups categorized as mild malarial anemia (MA), moderate MA, and severe MA [14].

Similarly, in many studies, bone marrow inhibition was found to be correlated with the degree of parasitemia and could be reversed after clearance of parasites from blood. In the in vitro study of fifteen bone marrow cultures of* P. falciparum* patients, abnormalities were observed only during parasitemia [15]. Premature death of normoblasts, decreased normoblastic number, and cellular iron incorporation and defective haemoglobin synthesis were observed during development of the cultures. Dormer et al. [16] analysed erythroblast cell kinetics in five cases of acute* P. falciparum* malaria and noted changes in erythroblast morphology and reduced rate of erythroblast proliferation while Abdalla and Wickramasinghe [17] observed wide variation in the number of BFUe (burst forming unit) and CFUe (colony forming unit) in the bone marrow of Gambian children with falciparum malaria and moderate or severe anaemia. Significantly lower number of BFUe were noted in the patients who had parasitemia >1%. Similarly, in the study involving young Gambian children with* P. falciparum* malaria, the children who presented with chronic anemia (parasitemia <1%) demonstrated higher levels of erythroid hyperplasia and dyserythropoiesis [18]. Verhoef et al. [19] studied 328 Kenyan children with asymptomatic malaria and evaluated erythropoiesis by serum concentrations of erythropoietin and soluble transferrin receptor. Lower haemoglobin and higher serum concentrations of erythropoietin and transferrin receptors were detected during the malaria infection. Conversely, disappearance of malarial antigenemia resulted in increased hemoglobin concentrations and decreased concentrations of these serum indicators.

#### 3.1.2. In Vivo Mice Studies

The above mentioned aspects of erythropoiesis were supported by the rodent malaria models which were proved to be useful in delineating the erythropoietic response followed by murine* Plasmodium* species. Several strains of* Plasmodium* can infect mice such as* P. chabaudi*,* P. berghei*,* P. yoelii,* and* P. vinckei*. Akin to the* P. falciparum*,* P. chabaudi* invades erythrocytes of all ages [20, 21] while* P. yoelii* has preference for reticulocytes [22] and hence may assist as a* P. vivax* model. Availability of differentially susceptible inbred mouse strains in which infections can be lethal and nonlethal improved the scope of murine models.

Maggio-Price et al. [23] experimentally infected rodents with* P. berghei* and attempted to characterize the erythropoietic response in terms of changes in marrow hematopoietic stem cells. Mice infected with* P. berghei* had dramatic decreases in bone marrow cellularity, erythroblasts, BFU-E, and CFU-E, 24 hours after infection. Similarly tissue culture studies of lethal (strain 17XL)* P. yoelii* infection in rodent showed decline in marrow BFU-E and marrow cellularity [24]. Erythropoietic responses during infection in resistant and susceptible mice were investigated during* P. chabaudi* AS infection [25]. It was observed that in vivo ^59^Fe incorporation was significantly more depressed in bone marrow and more increased in the spleen in resistant mice during the period of anemia. The increase in splenic ^59^Fe incorporation was a function of the size of the spleen. Chang and Stevenson [26] tried to investigate the mechanism of erythropoietic abnormalities by exploring upstream events of erythropoiesis affected by blood-stage* P. chabaudi* AS in mice treated with recombinant murine erythropoietin (EPO). It was found that suppression of EPO-induced proliferation of early EPOR-positive erythroid progenitors led to the impaired terminal maturation of TER119^+^ erythroblasts.

#### 3.1.3. In Vitro Culture Studies

In the recent years, after the successful production of erythrocytes from the in vitro cultures of haematopoietic stem cells [27], the model system was being employed in the elucidation of complexity of malarial anemia. The kinetics of differentiation in in vitro erythropoiesis model system closely resembles that of erythroid cell maturation in the bone marrow and differentiation occurs in a relatively synchronous manner.

Panichakul et al. [5] in 2012 reported the inhibition of erythroid cell expansion and differentiation followed by exposure of* P. vivax* infected intact erythrocytes as well as lysates in culture system established by isolating haematopoietic stem cells from normal human cord blood. Inhibition of erythroid development was determined by reduction in the expression of glycophorin A and CD71 on the growing erythroid cells. Similar observations were observed in the experiments performed with different laboratory strains of* P. falciparum* using the same culture system (unpublished observations).

Expression of CD71, essential for uptake of iron bound to transferrin, is normally increased on maturing erythroblasts that require iron from haemoglobin synthesis as well as on other actively growing cells [28]. These findings suggests that impaired erythroblast maturation may be a consequence of decreased iron uptake by developing RBCs. Additional studies are required to address this possibility.

#### 3.1.4. Gene Expression Studies

Transcriptional changes can be utilized as an important first step in understanding the host cell's adaptive response to infection by blood stage* Plasmodium*. Microarray analysis data of murine transcriptional responses during the infection revealed strongly suppressed erythropoiesis, starting early during infection, and highly upregulated transcription of genes that control host glycolysis, including lactate dehydrogenase [29]. Recently Tamez et al. [30] showed that exposure of* P. falciparum* to erythroid progenitors in vitro upregulated a set of genes which are associated with signalling, erythropoiesis, and erythroid cell development.

Gene expression profiling of growing erythrocytes could be an important key for understanding the pathophysiology of the disease. During the intraerythrocytic phase of its life cycle, malaria parasite matures within a cell in which haemoglobin is the single major cytosolic protein. Haemoglobin is the main amino acid reservoir available to the intraerythrocytic* Plasmodium*. The qualitative and quantitative changes in haemoglobin have been shown to inhibit the parasite growth in vitro [31, 32]. Being an important protein, it is important to check the expression profiling of globin genes which may help us to understand host parasite interactions and its potential contribution to both infection and disease.

Imbalance in *α* and *β* globin chains has been greatly discussed in cases of thalassemias [33–35]. Altered *α*/*β* globin gene expression ratio in such cases is an important indicator of ineffective erythropoiesis as well as disease severity [35, 36]. In 1975, Orkin et al. [37], working on murine erythroleukemic cell line, have reported differential expression of *α* and *β* globin genes during cellular differentiation. Substantial excess amount of *α* RNA was observed (*α*/*β* ratio ~3.7) early in induction, and the *α*/*β* RNA ratio progressively approached 1 as differentiation proceeds further. Studies are required to check if parasites or their products can affect the balance between productions of *α* and *β* globin chains in the erythroid progenitors. This extreme *α*/*β* anomaly may be the reason of severe ineffective erythropoiesis.

### 3.2. Factors Affecting Erythropoiesis

Various host and parasite mediators responsible for the erythropoietic changes during malaria infection have been documented in the literature, hemozoin crystals and cytokines being largely discussed.

#### 3.2.1. Malaria Pigment Hemozoin (Hz) and Hemozoin Generated Products

Hemozoin is a by-product of heme detoxification by malaria parasites through biocrystallization process. As the parasite multiplies in host, hemozoin is continuously produced and released together with the merozoites and engulfed by macrophages, monocytes, neutrophils, and other immune cells such as dendritic cells (DCs) [38]. Hemozoin stimulates the secretion of biologically active 4-hydroxy-nonenal (4-HNE) through oxidation of membrane lipids. It also activates macrophages and DCs to produce inflammatory cytokines [39]. Hemozoin and other generated products are receiving increasing attention due to their role in host immune system modulation.

Dysregulation in the innate immune response is believed to be an important cause of impaired erythroid responses in children with severe malarial anemia. The ability of* P. falciparum* derived Hz to cause dysregulation in pro- and anti-inflammatory cytokines, growth factors, chemokines, and effector molecules has been studied extensively [40].

Dysregulation of innate inflammatory mediators is a result of phagocytosis of malarial pigment hemozoin by immune cells. During acute falciparum malaria in children, altered production of soluble immune mediators, such as nitric oxide (NO) and prostaglandin-E2 (PGE2), has been observed from peripheral blood mononuclear cells (PBMCs) [41, 42]. Recent findings in a murine model of malaria demonstrate that injection of Hz in BALB/c mice induces the expression of chemokines (Macrophage Inflammatory Protein-1, MIP-1*α*, MIP-1*β*, MIP-2, and Monocyte Chemoattractant Protein-1, MCP-1) [43]. Moreover experiments using PBMCs from healthy, malaria-naive adults also illustrated dysregulation in *β*-chemokine production by Hz [44].

Hemozoin has been shown to inhibit the erythroid development in vitro and in vivo. Hemozoin within the bone marrow and plasma levels of hemozoin in patients with malarial anemia were associated with reduced reticulocyte response [45]. Conflicting results have been reported regarding inhibition of erythropoiesis with or without apoptosis by hemozoin crystals. Lamikanra et al. [45] described inhibition of erythroid cell development in vitro by* P. falciparum* isolated hemozoin independently of inflammatory mediators. It was characterised by delayed expression of the erythroid markers and increased apoptosis of progenitor cells. In the absence of tumor necrosis factor (TNF), hemozoin inhibited erythroid development in vitro. Conversely, after addition of TNF, it has been found to be synergized with hemozoin to inhibit erythropoiesis [46]. Giribaldi et al. [47] investigated the possible role of hemozoin and 4-hydroxynonenal in malarial dyserythropoiesis. 4-HNE generated by monocytes as well as supernatants of HZ and HZ-fed monocytes had been shown to inhibit progenitor growth. In the in vitro experiments performed by Skorokhod et al. [48], growth of erythroid cells was inhibited without apoptosis during cocultivation with HZ or treatment with low micromolar 4-HNE.

After HZ/HNE treatment, expression of cell-cycle regulation proteins was investigated and the expressions of critical proteins, p53 and p21, were increased while the master transcription factor in erythropoiesis, GATA-1, was found to be reduced. The regulator protein of G_1_-to-S-phase transition (retinoblastoma protein) was consequently hypophosphorylated. HZ and HNE inhibited protein expression of crucial receptors (R) in erythropoiesis, namely, transferrin R1, stem cell factor R, interleukin-3R, and erythropoietin R. Thus it was clear that HZ and HNE inhibited erythropoiesis by interfering with cell cycle and cell-cycle regulation proteins acted as targets for HZ and HNE in the inhibition process.

Hemozoin leads to the continuous targeting of the host innate immune system, leading to both pro- and anti-inflammatory responses; thus, currently, the potential application of hemozoin crystals for their use in vaccine as an adjuvant has been evaluated [49]. Both natural and synthetic forms of hemozoin are observed to possess adjuvant properties but used different innate immune receptors.

Further studies may provide deeper insights into the molecular mechanisms involved in immune responses to malarial infection.

#### 3.2.2. Cytokines

Severe disease in both human and mouse seems to be dependent on the levels of proinflammatory and anti-inflammatory cytokines. The relative balance between pro- and anti-inflammatory cytokines determines the degree of malarial anemia [50–54]. Higher levels of proinflammatory cytokines during the acute phase of infection appear to limit disease progression, while an anti-inflammatory response appears to promote enhanced pathogenesis [51, 55–57].

Parasites and its byproducts elevate a strong inflammatory response by increasing TNF*α* and IFN*γ*. These cytokines can inhibit all stages of erythropoiesis [58–60]. In a study involving* P. vinckei*, appreciable erythrophagocytosis and dyserythropoiesis were observed in bone marrow preparations from TNF-treated mice and those with severe illness due to* P. vinckei* [61].

The potent anti-inflammatory cytokine, IL10, has been suggested as important factor to regulate TNF*α* levels. The low IL10/TNF*α* ratio has been associated with severe anemia in young children [52, 62]. Many other proinflammatory cytokines such as IL12, nitric oxide (NO), and migration inhibitory factor (MIF) have been implicated in pathophysiology of anemia. IL12 is considered to be a stimulator of erythropoiesis [63, 64]. It is found to enhance the numbers of erythroid burst (BFU-E) and colony forming units (CFU-E) in bone marrow and spleen cells significantly in vitro from normal and day 7 infected resistance and susceptible mice. Effect of NO on cell development was studied in an in vitro model of erythropoiesis, developed using CD34+ stem cells derived from peripheral blood. NO significantly inhibited erythroid cell proliferation and maturation by increased apoptosis of erythropoietin-stimulated CD34+ cells [65].

#### 3.2.3. *β*-Chemokines

In addition to cytokines, the important mediators in malaria pathogenesis are chemokines. Chemokines are chemotactic cytokines having the ability to induce directed chemotaxis in nearby responsive cells. Chemokines are important for bridging innate and adaptive immune responses and regulating hematopoietic maturation [66, 67].

The chemokines expression profile has largely been studied for their potential role in regulating disease severity in malaria patients.

Ochiel et al. [44] determined circulating protein levels and transcript profiles of *β*-chemokines (Macrophage Inflammatory Protein 1 [MIP-1*α*, MIP-1*β*] and Regulated on Activation, Normal T-cell Expressed and Secreted [RANTES]) [68] and *α*-chemokine (Stromal cell Derived Factor 1 [SDF-1]) in plasma and PBMCs (peripheral blood mononuclear cells), respectively, in children with various degrees of* P. falciparum* malaria. Children with acute falciparum malaria showed dysregulation of *β*-chemokines characterized by elevated MIP-1*α* and MIP-1*β* and decreased RANTES at the mRNA and protein level. Significant reduction in circulating levels of RANTES was observed in healthy children with a history of severe malaria relative to those that previously experienced mild malaria indicating the protective role of RANTES against severe disease. Interestingly, transcriptional analysis of RANTES revealed that children with previous severe malaria had significantly higher RANTES mRNA expression than those with previous mild malaria. Similarly Were et al. [14] demonstrated the reduced circulating RANTES and peripheral blood mononuclear cell RANTES mRNA levels in Gabonese children with acute malaria (defined by hyperparasitemia and mild-to-moderate forms of anemia) [44].

RANTES is a specific chemoattractant for memory T cells and regulates inflammation by promoting leukocyte activation, angiogenesis, antimicrobial effects, and hematopoiesis [69]. It can promote migration of erythroid precursors into hematopoietic tissues [70] and prevent apoptosis of erythroid progenitors [71] suggesting that suppression of RANTES may lead to an ineffective erythropoietic response.

#### 3.2.4. Monocyte Migration Inhibitory Factor (MIF)

Ingestion of parasite infected erythrocytes or malarial pigment (hemozoin) induces the release of macrophage migration inhibitory factor (MIF) from macrophages, a proinflammatory mediator. MIF has been thought to have intrinsic role in the development of the anemic complications and bone marrow suppression that are associated with malaria infection. At concentrations found in the circulation of malaria infected patients, MIF was found to suppress erythropoietin-dependent erythroid colony formation. Moreover, MIF synergized with known antagonists of hematopoiesis, tumor necrosis factor, and *γ* interferon [72]. MIF also caused inhibition of erythroid (BFU-E), multipotential (CFU-GEMM), and granulocyte-macrophage (CFU-GM) progenitor-derived colony formation [73].

Mouse studies regarding MIF demonstrated similar observations. MIF was detected in the sera of* P. chabaudi* infected BALB/c mice, and circulating levels correlated with disease severity [73]. However, infection of MIF knockout mice with* P. chabaudi* resulted in less severe anemia, improved erythroid progenitor development, and increased survival compared with wild-type controls [72]. It is therefore conceivable that neutralization of MIF may protect against bone marrow suppression to some extent.

Consistent with the role of MIF as erythropoietic suppressor, the elevated serum MIF levels were found in patients with severe malaria [50]; however decreased serum concentration was observed in Kenyan children with malaria [74]. It was demonstrate that, in children, hemozoin acquisition by monocytes was associated with low levels of peripheral blood MIF and increased severity of anemia.

Soluble factors of* Plasmodium* have also been studied for their effect on erythropoiesis. Cell-free conditioned media prepared from spleen cells of mice infected with* P. chabaudi* [75] as well as* P. berghei* and* P. vinckei* [76] were able to inhibit erythroid cell proliferation of splenic erythroid cells in vitro and this inhibition was not reversed by increasing the concentration of EPO.

These factors either individually or collectively generate an ineffective erythropoietic response. Understanding the interplay between these mediators would provide the insights into mechanism invoking inadequate erythropoiesis.

## 4. Conclusion

Inadequate erythropoiesis is the important pathophysiology of malarial anemia. Understanding the pathophysiology and associated host parasite interactions would provide deeper insights into immune mechanism involved in the malarial infection and would help in the development of therapeutic strategies to treat severe malarial anemia. The interplay between various modulators causing ineffective erythropoiesis needs to be explored further.

It is also important to check whether parasites or their products mount any transcriptional responses in globin genes and affect the balance among the globin chains that can be analyzed for their potential contribution to the ineffective erythropoiesis and dyserythropoiesis.
